# FACTORS ASSOCIATED WITH RAPID WEIGHT GAIN IN PRESCHOOL CHILDREN IN PUBLIC DAY CARE CENTERS

**DOI:** 10.1590/1984-0462/;2018;36;3;00012

**Published:** 2018

**Authors:** Adriana de Sousa Nagahashi Lourenço, Daniela Almeida Neri, Tulio Konstantyner, Domingos Palma, Fernanda Luisa Ceragioli Oliveira

**Affiliations:** a Universidade Federal de São Paulo, São Paulo, SP, Brasil.

**Keywords:** Child daycare centers, Child nutrition, Weight gain, Obesity, Pré-escolar, Alimentação infantil, Ganho de peso, Obesidade

## Abstract

**Objective:**

To evaluate the prevalence, and identify factors associated with rapid weight gain in preschool children.

**Methods:**

A cross sectional study was carried out with 136 children between 24 and 35 months of age attending public daycare centers in Mogi das Cruzes between February and December 2014. Interviews were conducted with the mothers for clinical, sociodemographic and anthropometric characterizations of the children. It was considered to be rapid weight gain when the children presented a difference greater than 0.67 between the weight for age Z score from birth to evaluation. A logistic regression model was adjusted for factors associated with rapid weight gain.

**Results:**

Fifty children (36.8%) presented rapid weight gain and 36 (26.5%) were overweight. Of these, 22 children were in the rapid weight gain group. The logistic model showed that longest total breastfeeding time (OR 0.94, 95%CI 0.88-0.99; p=0.031) was a protection factor, and low socioeconomic level (OR 4.18, 95%CI 1.04-18.60; p=0.044) was a risk factor for rapid weight gain.

**Conclusions:**

There was a high prevalence of rapid weight gain and being overweight among three year old preschoolers attending public day care centers in Brazil. Encouraging the practice of breastfeeding children in the first years of life, in addition to giving guidance about appropriate foods, especially for families with a low socioeconomic status, can potentially contribute to reduce rapid weight gain and, consequently, future metabolic complications of being overweight.

## RESUMO

**Objetivo:**

Avaliar a prevalência e identificar os fatores associados ao ganho de peso rápido em pré-escolares.

**Métodos:**

Estudo transversal realizado entre fevereiro e dezembro de 2014, com 136 crianças entre 24 e 35 meses de idade frequentadoras de creches públicas do município de Mogi das Cruzes. Foram realizadas entrevistas com as mães para caracterização clínica, sociodemográfica e antropometria das crianças. Considerou-se ganho de peso rápido quando as crianças apresentaram diferença superior a 0,67 entre o escore Z de peso para idade do nascimento à avaliação. Foi ajustado modelo de regressão logística para fatores associados ao ganho de peso rápido.

**Resultados:**

Do total de crianças avaliadas, 50 (36,8%) apresentaram ganho de peso rápido e 36 (26,5%) excesso de peso. Destas, 22 crianças eram do grupo ganho de peso rápido. O maior tempo total do aleitamento materno (OR 0,94, IC95% 0,88-0,99; p=0,031) foi identificado como fator de proteção e o baixo nível socioeconômico (OR 4,18, IC95% 1,04-18,60; p=0,044), como fator de risco para ganho de peso rápido.

**Conclusões:**

Encontrou-se alta prevalência de ganho de peso rápido e de excesso de peso entre pré-escolares no terceiro ano de vida frequentadores de creches públicas. Incentivar a prática do aleitamento materno de crianças nos primeiros anos de vida e orientar a alimentação adequada, principalmente nas famílias de baixo nível socioeconômico, contribui potencialmente para a redução do ganho de peso rápido e, consequentemente, de futuras complicações metabólicas do excesso de peso.

## INTRODUCTION

A child’s growth after intrauterine life is influenced by several factors, especially maternal nutrition and the nutritional status of his or her first years of life, which may have an effect on metabolism programming,^1^ thus increasing the risk of chronic diseases.

Increasing numbers of infants have been enrolled in daycare centers in Brazil,^2^ because women are increasingly participating in the labor market, as a way to secure family income.^3^ This fact contributes to the low rates of Exclusive Breastfeeding (EB) and to the outsourcing of health care and food. In nurseries, infants receive about 70% of their daily nutritional needs.^4^ The growth and development of these children are influenced by the amount of time they attend daycare, the physical facilities, the knowledge and training of their caregivers, the plans for their diet, and the energy they expend on daily activities.^5^

An individual’s first two years of life, in conjunction with their gestational period, are very important stages. Thus, the first thousand days of life are fundamental for the control and prevention of chronic non-communicable diseases, like the risk of obesity in the short, medium and long term.^6^

Rapid weight gain (RWG) has been associated with being overweight in childhood and, consequently, with increased risks for metabolic syndrome and cardiovascular diseases.^7^ Ong et al. ^8^ ^,^ ^9^ defined RWG as an increase greater than 0.67 in the Z score of the anthropometric indicators of weight-for-age or weight-for-height, considering that these values are the amplitude between the percentile ranges on growth charts. The advantage of using these indicators in the postnatal phase allows for the early detection of risk and, consequently, the adoption of strategies to control and prevent nutritional disorders in childhood.^9^

Considering this context, the present study aimed to evaluate the prevalence, and to identify factors associated with RWG in preschool children attending public daycare centers.

## METHOD

A cross-sectional study was carried out between February and December 2014, with children between 24 and 35 months of age, who attend public daycare centers in Mogi das Cruzes, São Paulo, Brazil.

The municipality of Mogi das Cruzes has approximately 430,000 inhabitants,^10^ with an average per capita income of R$916.81 and a Municipal Human Development Index (MHDI) of 0.783.^11^. Children between 0 and 4 years old make up about 4% of the population. This municipality provides 4 daycare centers: 14 municipal units called Municipal Early Childhood Education Centers (*Centro de Educação Infantil Municipal* - CEIM) and 28 subsidized ones, which are administered by social entities or Non-Governmental Organizations (NGOs), with the support of the city hall.

Initially, one daycare center was selected for the pilot study. At this center, the field instruments were tested, in order to correct and adapt the data collection methodology.

Daycare centers were selected starting with schools administered exclusively by the city. Of the 14 CEIMs contacted, 13 accepted the proposal of receiving the field team. In order to provide proper representation of children enrolled in daycare centers, the total number of elementary schools in the municipality was considered.

The 13 daycare centers selected were classified according to the number of children enrolled. As such, 4 were considered to be small (less than 40 children), 7 were intermediate (between 40 and 60 children), and 2 were large (more than 60 children). The sample was selected so that there was at least one daycare center in each group. Since more than half of the daycare centers were intermediate, two daycare centers were selected in this category. Therefore, four daycare centers were included in the study: one small, two intermediate and one large. The daycare centers of each group were randomly selected with the same possibility of inclusion in the study. The sampling was carried out with the probability of selection of 1/4 for small daycares, 2/7 for intermediate and 1/2 for large ones.

In this regard, all 180 children between the ages of 24 and 35 months old who were regularly enrolled in the selected daycare centers were included. Additionally, 9 children that participated in the pilot study were included, totaling 189 children. Of these, 16 were excluded, as the parents or guardians did not authorize their participation in the research. Twenty-three were excluded due to lack of birth information. And 14 were excluded due to prematurity, which was considered when the birth occurred after less than 37 weeks of gestation,^12^ according to information collected from the child’s health card or, if the card was not available, from the person in charge. Thus, the final sample consisted of 136 children. This number of children (exposed and not exposed 1:1) is sufficient to identify odds ratios of 1.5 (alpha=0.05 and beta=0.20). The sample size was measured in the Epi-Info 2000 Program, version 3.4.3.^13^

Interviews were carried out with the mother or guardian of the child, through questionnaires with pre-coded questions regarding the gestational period, birth and the child’s current health. Additionally, information was collected from the child’s health card to supplement the information of interest.

An evaluation of feeding practices in the child’s first year of life included the classification and duration of breastfeeding, total time of breastfeeding, and the age that adequate solid foods (fruit, potatoes, and family meals) and unhealthy foods (processed foods) were introduced, using a food questionnaire developed for the study. Current consumption was estimated through the application of a food frequency questionnaire.^14^ The foods classified as processed were defined as unhealthy foods, according to the Food Guide for the Brazilian Population from the Ministry of Health.

The anthropometric measures measured were: height, weight, arm circumference (AC), triceps skinfold (TSF), subscapular skinfold (SS) and cephalic perimeter (CP), according to guidelines of the Brazilian Society of Pediatrics (*Sociedade Brasileira de Pediatria* - SBP).^16^ Height was measured using a portable Seca^®^ stadiometer, with measurements taken in millimeters. For weight, a Plenna^®^ digital anthropometric scale was used, with a scale of 0.1 kg and a maximum load of 150 kg. To measure AC and CP, an inelastic tape measure, measuring in millimeters was used. And for TSF and SS, a Lange^®^ brand adipometer was used. A previously trained researcher performed all of the field procedures, and all of the instruments were calibrated to avoid estimate errors.

To diagnosis nutritional status, criteria from the World Health Organization (WHO) from 2006 were used, according to the classification of anthropometric indexes for the weight-for-age (ZW/ A), height-for-age (ZH/A), weight-for-height (ZW/ H) and body mass index for age (Z-BMI/A) Z scores. A child was considered to overweight when the Z-W/H score was above +1. The body composition (percentage of lean and fat mass) was evaluated using anthropometric measurements (AC and TSF / SS). The children were classified as being in the RWG group when the difference between the Z-W / A of the evaluation and the Z-W/A of the birth was > 0.67.^7^ ^,^ ^8^ ^,^ ^9^

To make a socioeconomic classification, an instrument proposed by the Brazilian Association of Research Companies (*Associação Brasileira de Empresas de Pesquisa* - ABEP),^17^ was used, which measures the purchasing power and the level of schooling of the head of the family, in addition to living conditions.

The distribution of variables was assessed using the Shapiro Wilk test. Continuous variables were described using median and interquartile range and categorical variables were described in absolute and percentage distribution. The differences between the means of the continuous variables were evaluated using Student’s t test or Mann Whitney test, according to the distribution of the variable. The differences between the categorical variables were assessed according to the chi-square test. A multiple logistic regression analysis was performed in order to identify the factors associated with RWG and to control confounding variables. The criterion used for inclusion in the model was a p-value≤0.20 in the univariate analysis. A plausible effect of complementary feeding on RWG was controlled in the multiple model, with the use of the variable “age solid foods were introduced”. The input method of the variables was the stepwise forward method. For all other analyses, p was considered to be significant at a level of less than or equal to 0.05. The quality of fit of the model was verified using the Hosmer Lemeshow test. Statistical analyses were performed using the SAS-JMP program (Pro 10 Version) (SAS Institute, Cary, NC).

The study was submitted and approved by the Research Ethics Committee of the Paulista School of Medicine of the Universidade Federal de São Paulo (EPM / UNIFESP), under the report number 415.648.

## RESULTS

Of the 136 children evaluated, 50 (36.8%) presented RWG from birth until the date of collection. Table 1 shows the maternal, neonatal and socioeconomic characteristics of the population according to the presence or absence of RWG. The groups were similar in relation to age and gender. However, children with RWG had lower values of gestational age, weight, length and anthropometric indices at birth, when compared to those without RWG.

**Table 1:** Maternal characteristics and child characteristics (n=136), according to rapid weight gain or no rapid weight gain in public daycare centers in Mogi das Cruzes, 2014.

	Total Median (P25; P75) or n (%) (n=136)	RWG^a^ Median (P25; P75) or n (%) (n=50)	NRWG Median (P25; P75) or n (%) (n=86)	p-value*
Maternal				
Age (years)^c^	27 (21.0; 30.0)	26.5 (21.7; 30.2)	27 (20.0; 30.0)	0.920
Age <25 years old^c^	46 (36.2)	13 (28.2)	33 (40.7)	0.150
Smoked during pregnancy^b^	14 (10.4)	8 (16.0)	6 (7.0)	0.090
Drank alcohol during pregnancy^b^	21 (15.6)	10 (20.0)	11 (13.0)	0.200
Level of schooling (≤8 years of study)^b^	25 (18.4)	12 (24.0)	13 (15.1)	0.200
Social level (C2 e D)^b^	20 (14.7)	10 (20.0)	10 (11.6)	0.180
Children				
Age when started to go to daycare (months)	12 (6.0; 18.0)	12 (6.0; 19.2)	9 (6.0; 18.0)	0.456^‡^
Gestational age (weeks)	39 (38.0; 40.0)	38 (38.0; 39.0)	39 (38.0; 40.0)	0.008
Age (months)	30 (27.0; 34.0)	30 (25.7; 34.0)	30 (27.0; 33.0)	0.980
Female gender^b^	69 (50.7)	28 (56.0)	41 (48.0)	0.350
Anthropometric measures and indices at birth				
Weight (kg)	3.3 (2.9; 3.6)	2.9 (2.6; 3.2)	3.5 (3.2; 3.7)	<0.001
Height (cm)^d^	49 (48.0; 51.0)	48 (47.0; 50.0)	50 (48.0; 51.0)	0.003
Z-W/A^e^	0.05 (-0.72; 0.60)	-0.71 (-1.42; -0.22)	0.38 (-0.23; 0.84)	<0.001
Z-H/A^d,e^	-0.35 (-1.00; 0.59)	-0.67 (-1.15; 0.26)	0.06 (-1.00; 0.59)	0.005
Z-W/H^d,e^	0.26 (-0.67; 0.96)	-0.55 (-1.15; 0.3)	0.52 (-0.10; 1.42)	<0.001
Z-BMI/A^d,e^	0.10 (-0.60; 0.73)	-0.71 (-1.29; 0.05)	0.50 (-0.14; 1.09)	<0.001

RWG: rapid weight gain; NRWG: no rapid weight gain; P25; first quartile; P75: third quartile; Z-W/A: weight-to-age Z score; Z-H/A: height-to-age Z score; Z-W/H: weight-to-height Z score; Z-BMI/A: Body Mass Index-to-Age Z score; ^a^increase in weight-for-age Z score >0.67 from birth to the time of evaluation; ^b^n (%); ^c^n=127 complete data; ^d^n=127 with complete birth height data (RWG group, n=44; NRWG group, n=83); ^e^WHO, 2006;*p-value based on Student’s t-test; ^‡^p-value based on Mann-Whitney’s test.

Table 2 shows the anthropometric data at the time of the study according to RWG. Note that children who gained weight quickly had a Z score of the highest anthropometric indexes, including for body composition measurements. The prevalence of overweight children (Z-W/ H> 1) was 26.5%, with a statistically significant difference between RWG and non-RWG groups (44.0 versus 16.3%, p<0.001). The median weight gain between birth and the time of evaluation for the total population was 10.3 kg, also with difference between groups (11.2 and 9.8 kg, p=0.001).

**Table 2:** Anthropometric evaluation of the children (n=136), according to rapid weight gain or no rapid weight gain in public daycare centers in Mogi das Cruzes, 2014.

	Total Median (P25; P75) or n (%) (n=136)	RWG^a^ Median (P25; P75) or n (%) (n=50)	NRWG Median (P25; P75) or n (%) (n=86)	p-value
Age (months)	30 (27.0; 34.0)	30 (25.7; 34.0)	30 (27.0; 33.0)	08971^‡^
Measurements				
Weight (kg)	13.7 (12.5; 14.7)	14.1 (13.3; 15.8)	13.3 (12.0; 14.3)	<0.001^‡^
Height (cm)	91.4 (88.0; 94.1)	91.7 (89.8; 95.6)	91.0 (87.4; 93.5)	0.093*
CP (cm)	49.2 (48.5; 50.3)	49.5 (48.7; 50.2)	49 (48.0; 50.3)	0.123*
AC (cm)	16.4 (15.5; 17.0)	16.9 (16.0; 17.5)	16.0 (15.0; 16.7)	<0.001^‡^
TSF (mm)	10.0 (9.0; 12.0)	11.0 (9.0; 13.0)	10.0 (9.0; 11.7)	0.002^‡^
SS (mm)	6.0 (5.0; 7.0)	6.0 (5.0; 8.0)	6.0 (5.0; 7.0)	0.150^‡^
Anthropomorphic indices				
Z-W/A^d^	0.29 (-0.42; 1.02)	0.79 (0.01; 1.39)	0.03 (-0.58; 0.63)	<0.001*
Z-H/A^d^	-0.22 (-0.75; 0.64)	0.43 (-0.43; 1.11)	-0.30 (-0.81; 0.28)	<0.001*
Z-W/H^d^	0.49 (-0.20; 1.02)	0.92 (0.09; 1.38)	0.20 (-0.31; 0.78)	0.001*
Z-BMI/A^d^	0.47 (-0.09; 1.11)	0.93 (0.11; 1.36)	0.21 (-0.24; 0.91)	0.003*
Z-CP^d^	0.59 (-0.06; 1.16)	0.76 (0.33; 1.32)	0.55 (-0.26; 0.97)	0.034*
Z-AC^d^	0.63 (-0.13; 1.29)	1.03 (0.49; 1.56)	0.46 (-0.32; 1.03)	<0.001^‡^
Z-TSF^d^	1.13 (0.63; 1.89)	1.29 (0.69; 2.25)	1.05 (0.39; 1.72)	0.008*
Z-SS^d^	-0.05 (-0.94; 0.93)	0.10 (-0.92; 1.56)	-0.06 (-0.96; 0.92)	0.283^‡^
Z-W/H >+1^b,d^	36 (26.5)	22 (44.0)	14 (16.3)	<0.001^†^
Weight gain (P^c^-Pn)(kg)	10.3 (9.4; 11.1)	11.2 (10.4; 12.9)	9.8 (8.8; 10.6)	<0.001
Delta Z-P/I (Z-P/I^c^ -Z-P/In)^d^	0.28 (-0.43; 1.13)	1.43 (1.11; 1.96)	-0.22 (-0.91; 0.20)	0.958*

RWG: rapid weight gain; NRWG: no rapid weight gain; P25; first quartile; P75: third quartile; CP: cephalic perimeter; AC: arm circumference; TSF: triceps skinfold; SS: subscapular skinfold; Z-W / A: weight-for-age Z score; Z-H / A: height-for-age Z score; Z-W / A: weight for height Z score; Z-BMI / A: body mass index-for-age Z score; Z-CP: cephalic perimeter Z-score; Z-AC: arm circumference Z score; Z-TSF: tricipital skinfold Z score; Z-SS: subscapular skinfold Z score; W: weight; Wn: birth weight; Z-W/ In: weight-for-age Z score at birth; ^a^ increase in weight-for-age Z score > 0.67 from birth to time of the study; ^b^n (%); weight-for-age Z score at the time of study; ^d^WHO, 2006; * p-value based on Student’s t-test; ^‡^p-value based on the Mann Whitney test; † p-value based on the chi-square test.

In the evaluation of feeding practices (Table 3), no differences were found between the groups in relation to breastfeeding (exclusive or predominant), the premature introduction of potatoes into the child’s diet, and the frequent consumption of unhealthy foods. However, total breastfeeding time was higher in the non-RWG group compared to the RWG group (7 months versus 6 months, p = 0.01).

**Table 3:** Children’s (n=136) dietary practices, according to rapid weight gain or no rapid weight gain, in public daycare centers of Mogi das Cruzes, 2014.

	Total Median (P25; P75) or n (%) (n=136)	RWG^a^ Median (P25; P75) or n (%) (n=50)	NRWG Median (P25; P75) or n (%) (n=86)	p-value*
Duration of breast feeding (months)				
Exclusive^b^	3 (2; 5)	3 (2; 4.5)	4 (3; 5)	0.200
<6 months^b.c^	92 (79.2)	33 (80.5)	59 (78.7)	0.690^†^
Predominate	4 (3; 6)	4 (3; 6)	4 (3; 6)	0.140
Total	6(4; 14)	6 (3; 9)	7 (4; 20)	0.010
Age solid foods were introduced (months)^d^	5 (4; 6)	6 (4; 6)	5 (4; 6)	0.335
Consumption frequency of unhealthy foods (two or more times / day)^c.e^	65 (51.6)	25 (55.5)	40 (49.4)	0.500^†^

RWG: rapid weight gain; NRWG: no rapid weight gain; P25; first quartile; P75: third quartile; ^a^ increase in weight-for-age Z score > 0.67 from birth to the time of the study; ^b^n=116 received exclusive breastfeeding (rapid growth group. n=41; non-rapid growth group. n=75). 14 did not receive exclusive breastfeeding and 6 did not have data; ^c^n (%); ^d^n=127 with complete data for the age when solid foods were introduced (RWG group. n=45; NRWG group. n=82); ^e^n=126 with complete data on the consumption frequency of unhealthy foods (RWG group. n=45; NRWG group. n=81); *p-value based on Student t’s test; ^†^p value based on the chi-square test.

Table 4 shows the crude and adjusted analyzes of factors associated with RWG. It was observed that highest total breastfeeding time was associated with a lower risk of RWG (Odds Ratio - OR 0.94, 95% confidence interval - 95%CI 0.88-0.99, p=0.031). In addition, children of lower social classes (socioeconomic levels C2 and D) had a higher risk of RWG (OR 4.18, 95%CI 1.04-1.8.60, p=0.044) than those of higher classes (socioeconomic levels A, B and C1). These risk conditions were identified after adjusting the multiple model for birth weight, height, age, and age solid foods were introduced. The Hosmer Lemeshow test showed that the model conforms to the data (Goodness of fit: p=0.267).

**Table 4:** Factors associated with rapid weight gain* in the second year of life of children (n=117) attending public daycare centers in Mogi das Cruzes. 2014.

Factors	Univariate		Multivariate	
	OR (95%CI)	p-value	AOR (95%CI)	p-value
Total breastfeeding duration (months)	0.94 (0.89-0.98)	0.011	0.94 (0.88-0.99)	0.031
Social class (C2 and D)	1.90 (0.19-0.72)	0.190	4.18 (1.04-18.60)	0.044

Model adjusted for birth weight and height. age of child and age solid foods were introduced. OR: Odds Ratio; 95%CI: 95% confidence interval; AOR: Adjusted odds ratio; ^*^increase in weight-for-age Z score > 0.67 from birth to the moment of the study; Goodness-of-fit: p=0.2667.

## DISCUSSION

The prevalence of being overweight and RWG was 26.5 and 36.8%, respectively. Gestational age, total breastfeeding time, and anthropometric measures and indices showed statistically significant differences in the bivariate analysis, when the groups with and without RWG were compared. In addition, a short total amount of breastfeeding time and low socioeconomic levels were associated with a higher risk of RWG, regardless of the age of the child, the age solid foods were introduced, and weight and length at birth.

The children of the RWG group had lower anthropometric measures and indices at birth than those in the RWG group. Conversely, at the time of the study, the RWG group presented higher values of these measures and indices. The RWG indicator is related to Barker’s fetal origin hypothesis, which states that fetal malnutrition and, consequently, inadequate child growth is associated with the development of chronic diseases such as obesity and cardiovascular diseases.^18^ In addition, rapid growth in postnatal life, influenced by an obesogenic food environment and by interactions between nutrients and the genetic characteristics of individuals, favors the development of these diseases in adult life.^6^

In this regard, Programming, which consists of the induction, deletion or impairment of the development of a somatic structure or adjustment to a physiological system by a stimulus or aggression in the early stages of life, may lead to impairments of physiological functions and contribute to the disease. Thus, the affected gene expression may result in changes in eating behavior and, consequently, metabolic dysfunctions in adipose and muscle tissues, in the liver and in pancreatic β cells.^6^

In the evaluation of feeding practices, 20.7% of the children received EB for up to six months, which was lower than the national prevalence of 39.8% - found in the National Demographic and Health Survey/2006 (*Pesquisa Nacional de Demografia e Saúde* - PNDS) - and the worldwide prevalence of 34.8%.^19^ ^,^ ^20^ Such a difference may be related to the low socioeconomic status of the families being studied. In spite of this, the median duration of 3 months of EB demonstrated in this study was higher than that observed in the Brazilian capitals and the Federal District in the national health survey conducted in 2009 (1.8 months).^21^ This contrast can be explained by the kinds of mothers studied - mothers who need to return to work after their maternity leave period. Additionally, it can be explained by the pre-requisites established for enrolling children in daycare centers, which allows the mother to be employed.

With regard to the prevalence and the factors associated with RWG, Ong et al..^22^ found that 30.7% of infants with RWG had lower birth weight and height values and a higher percentage of primiparous mothers and mothers who smoked during pregnancy, when compared to the children in the control group. Although these associations have also been described in other studies, the present study did not identify these situations as factors associated with RWG in the multiple analysis.^7^ ^,^ ^23^ ^,^ ^24^

However, the children in the RWG group received less total breastfeeding time when compared to the RWG group (median: 6 months *versus* 7 months, p=0.001). This difference between groups, regardless of birth weight and height, child’s age and the age solid foods were introduced, may indicate the protective role of breast milk in the first months of life. From the moment the child stops receiving breast milk, other foods are introduced which do not always meet their nutritional needs, and often exceed the recommended energy consumption. This excess, in turn, increases the risk of RWG, especially in the stage of life in which the nutritional status is highly sensitive to changes in food consumption.^8^ ^,^ ^9^ ^,^ ^25^

Thus, the results of the present study demonstrate that with each month of breast milk consumption there is a 6% less risk of accelerated weight gain. In fact, studies have shown that the longer children breastfeed, the more of a protective effect it provides in relation to being overweight in childhood.^26^ ^,^ ^27^ One of the benefits of breast milk is its composition, which has high protein quality in low quantities. Excessive intake of protein, especially branched amino acids such as leucine, in the first two years of life is associated with an increased risk of adiposity, demonstrating that, in addition to the amount of protein consumed by the infant, the type of protein offered and the differences in the amino acid composition are important in order to protect him or her from the risk of becoming overweight.^28^ In addition, the child receiving breastfeeding often self-regulates their appetite more effectively and is therefore less susceptible to establishing strict eating times and to consuming certain amounts of milk.

Furthermore, children from families of lower socioeconomic classes were at a higher risk of being in the RWG group. Although there is no evidence in the literature between the association of socioeconomic status with this specific outcome, there are numerous studies that have demonstrated an association between low socioeconomic status with becoming overweight as a result of easy access to processed, high energy density and low nutritional value foods.^9^

This result corroborates what has been found in developed countries, where being overweight has been associated with worse socioeconomic conditions.^29^ This fact is reinforced by how socioeconomic classification was determined in this study. It considers the possession of goods and of the level of schooling of the head of household. Therefore, our findings confirm those found in other studies, which observed a negative association between maternal schooling and family income with unhealthy food consumption.^30^ However, there are Brazilian studies that suggest that the prevalence of being overweight is higher in children belonging to higher socioeconomic classes, which have more purchasing power for goods and food.^31^ This divergence can be explained by the diversity of the population of Brazilian children living at different socioeconomic levels, and by the specificity of the population of children attending daycare centers.

Maternal education also appears to influence the quality of infant feeding and nutrition. People’s knowledge about food is usually acquired through the radio, television, magazines and newspapers, which strongly influences these household’s food choices.^30^ Mothers who use formulas for children are more likely not to follow all of the recommendations with regard to complementary feeding.^32^ What seems sensible to consider is that, in some way, the child’s socioeconomic level and the parents’ schooling can interfere in their nutritional status, and there is a multi-causality characteristic of the being overweight which is particular to each family or population group.

Nevertheless, although it is not possible to say that every child with RWG during this phase will be overweight, because it is a multifactorial nutritional disorder, it seems important to investigate, monitor and guide the dietary intake of this group of children.

It should be noted that the population studied was socioeconomically homogeneous and had specific healthcare and educational characteristics, because they attend public and philanthropic daycare centers. However, about 1/4 of the population studied was overweight during their third year of life, demonstrating the risk of obesity. An extrapolation of the results presented in this study to children of other characteristics should be performed carefully, despite the fact that the findings confirm the need for weight monitoring in the early years of life. In addition, understanding the importance for children to receive high quality food during their first thousand days of life for neuroendocrine regulation and for their body composition, our questionnaire, which focused on introducing food and feeding frequency may not have accurately reported on food consumption and when the investigated foods were introduced. This could have been minimized if a validated instrument were available to assess the consumption of processed foods. Other limitations of the present study were sample size and the low explanatory power of the multiple model, which identified only two factors associated with RWG. This evidence points to the existence of other factors not identified in this investigation, which proves the multifactorial characteristic of determining RWG in children in the studied age group.

A longer period of time for breastfeeding proved to be a protective factor for RWG, and the low socioeconomic status of the families was associated with a higher risk of Z-W/A evolution. Therefore, RWG identification strategies and early nutritional therapeutic measures should be performed to prevent and control children from being overweight. Incentives for breastfeeding and the improvement of the socioeconomic conditions of the populations at risk are important. However, there are many challenges with regard to the improvement of these conditions on a large scale in developing countries, such as Brazil, and the change in food behavior in the populations with low incomes and low levels of schooling. These challenges range from planning to direct actions made by pediatricians and other health professionals, who propose changes in the attitudes of parents that are responsible for feeding their child and set an example for forming eating habits.

Thus, promoting extended maternal breastfeeding, developing nutritional and educational strategies, monitoring nutrition in children’s’ first years of life, and creating possibilities for improved family life conditions may contribute to the reduction of postnatal excess weight and its future metabolic complications. It is recommended that additional studies be developed with more detailed information on dietary patterns, using validated indicators of processed foods consumption in children with other socioeconomic/cultural characteristics to determine new interventions and to identify other factors associated with RWG and future obesity.
